# Biochemical, Molecular, and Transcriptional Highlights of the Biosynthesis of an Effective Biosurfactant Produced by *Bacillus safensis* PHA3, a Petroleum-Dwelling Bacteria

**DOI:** 10.3389/fmicb.2017.00077

**Published:** 2017-01-25

**Authors:** Abdulsamie Hanano, Mouhnad Shaban, Ibrahem Almousally

**Affiliations:** Department of Molecular Biology and Biotechnology, Atomic Energy Commission of SyriaDamascus, Syria

**Keywords:** petroleum crude oil, biosurfactant, glycolipid, *Bacillus safensis* PHA3, β-diglucosyldiacylglycerol synthase (β-DGS)

## Abstract

Petroleum crude oil (PCO)-dwelling microorganisms have exceptional biological capabilities to tolerate the toxicity of petroleum contaminants and are therefore promising emulsifier and/or degraders of PCO. This study describes a set of PCO-inhabiting bacterial species, one of which, identified as *Bacillus safensis* PHA3, produces an efficient biosurfactant which was characterized as a glycolipid. Fourier transform infrared spectrometer, nuclear magnetic resonance, Thin layer chromatography, HPLC, and GC-MS analysis of the purified biosurfactant revealed that the extracted molecule under investigation is likely a mannolipid molecule with a hydrophilic part as mannose and a hydrophobic part as hexadecanoic acid (C16:0). The data reveal that: (i) PHA3 is a potential producer of biosurfactant (9.8 ± 0.5 mg mL^-1^); (ii) pre-adding 0.15% of the purified glycolipid enhanced the degradation of PCO by approximately 2.5-fold; (iii) the highest emulsifying activity of biosurfactant was found against the PCO and the lowest was against the naphthalene; (iv) the optimal PCO-emulsifying activity was found at 30–60°C, pH 8 and a high salinity. An orthologous gene encodes a putative β-diglucosyldiacylglycerol synthase (β-DGS) was identified in PHA3 and its transcripts were significantly up-regulated by exogenous PAHs, i.e., pyrene and benzo(e)pyrene but much less by mid-chain *n*-alkanes (ALKs) and fatty acids. Subsequently, the accumulation of β*-DGS* transcripts coincided with an optimal growth of bacteria and a maximal accumulation of the biosurfactant. Of particular interest, we found that PHA3 actively catalyzed the degradation of PAHs notably the pyrene and benzo(e)pyrene but was much less effective in the mono-terminal oxidation of ALKs. Such characteristics make *Bacillus safensis* PHA3 a promising model for enhanced microbial oil recovery and environmental remediation.

## Introduction

For the purpose of environmental protection, governments and international organizations have enacted strict regulations for controlling the process of overexploitation and possibly inappropriate release of PCO throughout the world (UN, 2008). While these regulations have effectively contributed to minimizing the pollution of environment by the PCO, they can unfortunately be relatively ineffective in the face of contamination that occurs accidentally or unintentionally. Whatever the causes, when the pollution by the PCO occurs, multiple and diverse ecotoxicological effects are produced in both terrestrial and aquatic environments (Essien et al., 2015). The application of physical methods for removing the PCO from the aquatic environment can be a difficult, expensive and inefficient process, and the use of chemical surfactants as remediating agents is no longer favored because of their toxic effects on the biota of targeted area (Perfumo et al., 2010; Zdarta et al., 2016).

Therefore, bioremediation, defined as the use of microorganisms to degrade pollutants owing to their diverse metabolic capabilities, is considered as an evolving method for the degradation and removing of many environmental pollutants including hydrocarbons (Medina-Bellver et al., 2005; Ron and Rosenberg, 2014). The biodegradation of PCO by microorganisms has been the subject of many excellent reviews during the past decade (Hamamura et al., 2006; Kanaly and Harayama, 2010; Silva et al., 2014; Varjani, 2017). A considerable number of investigations has reported that bacteria are the most active microorganisms in crude oil degradation, and several bacteria are even known to feed exclusively on hydrocarbons (Acosta-Gonzalez and Marques, 2016; Varjani, 2017). Other organisms, including some archaea and a few yeast genera, namely, *Candida, Yarrowia, Pichia*, and more recently *Saccharomyces* are also described as powerful degraders of hydrocarbons and more particularly the ALKs (Iida et al., 2000; Hanano et al., 2013).

Due to their high hydrophobicity, hydrocarbons are insoluble in water and their bioavailability is therefore a first challenge to be surpassed by biodegraders. The hydrophobic organic compounds degrading microorganisms overcome this challenge by producing biosurfactants that ensure the emulsification of such hydrophobic compounds (Chandankere et al., 2013; Gargouri et al., 2016; Golshan et al., 2016; Ndlovu et al., 2016). The biosurfactants are a heterogeneous group of surface-active amphiphilic molecules produced by microorganisms for reducing interfacial tension between the aqueous and the organic phases resulting in the bioavailability of target compounds. Based on their chemical composition and microbial origin, biosurfactants can be classified as glycolipids, lipopeptides, fatty acids, and others (Gautam and Tyagi, 2006; Thavasi et al., 2011a; Bezza and Chirwa, 2017). It was reported that biosurfactants can replace the chemically synthesized surface-active agents in multiple applications and especially in environmental one (Singh et al., 2007; Sivasankar and Suresh Kumar, 2017). Even more, they gained, in two last decades, more attention as promising alternatives to chemicals in the respect of their biodegradability, better environmental compatibility, lower toxicity, high specific activity at extreme temperatures, pH, and salinities (Desai and Banat, 1997; Bezza and Chirwa, 2016; Jemil et al., 2016). Microorganisms, dwelling in the PCO, have exceptional biological capabilities to tolerate the toxicity of this complex and they therefore presumed to be promising emulsifier-degraders of PCO in the aquatic environments. Following this logic, the main purpose of the current work was to isolate, if existed, the PCO-inhabiting microorganisms in the objective of their identification, characterization and studying their possible potentiality as emulsifier-degraders of the PCO. Seven bacterial strains were therefore purely isolated and one of them was selected by its remarkable ability to consume the PCO. The bacterial isolate, identified as *Bacillus safensis* PHA3, produces an efficient biosurfactant for initiating the biodegradation of PCO. Therefore, the biochemical nature of the biosurfactant was determined and the biochemical, molecular, and transcriptional aspects that regulate its biosynthesis were highlighted. The outcome of this work will support our efforts to identify new microorganisms with promising activities in removing the petroleum-origin pollutants from the aquatic environment.

## Materials and Methods

### Petroleum Crude Oil, Culture Conditions, and PCO-Inhabiting Bacterium Isolation

Petroleum crude oil (light brown; pH, 5.2) was obtained from petroleum reservoir for oil field (TANAK), located in the north eastern region of Syria. PCO-inhabiting microorganisms were isolated by applying enrichment culture technique using minimal salt medium (MSM). The composition of MSM medium was as described before (Hanano et al., 2014). PCO (2%) (v/v) was used as a sole source of carbon in MSM and incubated at 28°C on a rotary shaker at 200 rpm for 7 days. Following the incubation period, 1 mL of culture was transferred to fresh medium containing 2% of PCO (v/v) and re-incubated for another 7 days. During all cycles of enrichment, salinity was maintained by adding NaCl (30 g L^-1^) in MSM. Following consecutive five cycles of enrichment, 1 mL of culture was diluted and plated on solidified MSM with agar. The grown bacterial colonies were further purified on Luria-Bertani (LB) agar plates containing (g L^-1^): peptone 10.0, NaCl 5.0 and yeast extract 10.0. The isolated strains were stored as frozen stock cultures at -80°C in 25% (v/v) glycerol. All chemicals, including ALKs and fatty acid standards, are purchased from Merck & Co. USA.

### 16S rDNA Sequencing, Phylogenetic Analysis, and Scanning Electron Microscopy

Genomic DNA was extracted from the bacterial isolates as previously described. Fragments about of 1500 bp of the *16S rRNA* genes were amplified using the universal primers 27F and 1492R (Supplementary Table S1). The PCR amplifications were performed as described before. The amplified *16S rRNA* encoding genes were sequenced by an ABI 310 Genetic Analyzer (Applied Biosystems). The *16S rRNA* gene sequences were analyzed against the related sequences in the GenBank-NCBI database and using the BLAST search program. The genus or species of the bacterial isolates were therefore identified. The *16S rRNA* sequences reported in this study have been submitted to the GenBank-NCBI under the accession numbers presented in **Table 1**. Phylogenetic analysis was carried out based on the score of blasting between *16S rRNA* sequences using http://blast.ncbi.nlm.nih.gov/Blast.cgi. Phylogenetic tree was constructed online using http://www.phylogeny.fr. For Scanning Electron Microscope (SEM) imaging, the bacterial cells were taken from a 6 days-old culture in MSM + 2% PCO, washed twice with MSM, spread on an aluminum disk and left inside a warm chamber (45°C) until a complete dryness. A SEM imaging was performed using a Vega II XMU system (TESCAN, Czech).

**Table 1:** Molecular identification of the PCO-inhabiting bacterial isolates.

PCO-inhabiting Isolates	*16S rRNA* homology (More than 99%)	*16S rRNA* GenBank Accession N.	Major characteristics
PHA2	*Bacillus pumilus*	KF267747	High resistance to environmental stresses (Kempf et al., 2005).
PHA3	*Bacillus safensis*	KF267748	Tolerance to spacecraft associated environments (Satomi et al., 2006).
PHA5	*Planomicrobium* sp.	KF267750	Antarctic Sea ice-dwelling (Junge et al., 1998).
PHA6	*Psychrobacter* sp.	KF267751	Support a wide range of cold and salinity (Kim et al., 2012).
PHA7	*Bacillus licheniformis*	KF267752	Commonly found in soil and bird feathers (Lin et al., 1992).
PHA8	*Streptomyces champavatii*	KF267753	Antibiotic production (Milind et al., 2001).
PHA9	*Kocuria flava*	KF267754	Degradation PAHs (Zubair et al., 2010).

### Physico-Chemical Screening for the Biosurfactant Producer Bacteria

The bacteria was tested for its potential to produce biosurfactant by using a hemolytic assay as Mulligan et al. (1984) reported. The oil-displacement test was done as described by Rodrigues et al. (2006). For hydrocarbon overlay agar (HOA) test, MSM agar plate was covered with 100 mL of PCO. A pure bacterial isolate was spotted as a single point on the surface of the PCO-coated plate. Plate was incubated for 48 h at 30°C. Colony surrounded by an emulsified halo was considered positive for biosurfactant production. Cell hydrophobicity was measured by BATH according to Rosenberg et al. (1980). The optical density (OD) of the aqueous phase was then measured at 600 nm in a spectrophotometer (VIS 6315, JENWAY, UK). For a given sample, three independent determinations were made and the mean value was taken.

### Time Course, Extraction, Purification, and Biochemical Analysis of the Biosurfactant

The production was carried out in 500 mL Erlenmeyer flasks containing 250 mL of MSM medium supplemented with 2% (v/v) crude oil as a unique carbon source. Each flask was inoculated with 2 mL of pre-cultured bacteria and incubated at 28°C at 200 rpm for 14 days. Bacterial cell growth was estimated using viable cell count method and expressed as Log CFU mL^-1^. Time course samples of culture medium were drawn daily and monitored for biosurfactant production as a function of the bacterial cell growth. The extraction of biosurfactant was carried out as previously described by Aparna et al. (2012) and its purification was performed into silica gel (60–120 mesh) glass column with chilled acetone as an eluent. The eluted fractions was pooled and the solvent was evaporated to dryness. Finally, the purified fraction was dissolved in a sterile-distilled water and conserved at 4°C until further characterization. Carbohydrate content of the biosurfactant was determined by the phenol–sulfuric acid method (Dubois et al., 1956). Protein content was determined by the method of Lowry et al. (1951) and lipid content was estimated according to Folch et al. (1957).

### Fourier Transform Infrared Spectrometer (FTIR), NMR, Thin Layer Chromatography (TLC), HPLC, and GC-MS Analysis

The infrared spectra of the extracted biosurfactant was determined using a Fourier transform infrared spectrometer (FTIR) (Thermo – Nicolet 6700 FTIR spectrometer equipment, Boston, MA, USA) as described before (Hanano et al., 2015). The structure of the purified glycolipid was characterized by^1^H- and ^13^C-NMR with a Varian INOVA 400 (400 MHz) at 30°C using CDCl_3_ solution. Both proton and carbon NMR chemical shifts were stated in ppm relative to the solvent shift as chemical standard. For further analysis of sugar moiety, purified biosurfactant was acid-hydrolyzed according to Al-Fadhli et al. (2006). In brief, 5 mg of pure biosurfactant in 5 mL of 2% H_2_SO_4_ in methanol was refluxed for 3 h followed by the addition of 4 mL of water to the reaction mixture. Methanol was evaporated and the aqueous solution extracted with chloroform and then neutralized with barium hydroxide. Precipitated barium sulfate was filtered through SiO_2_ (Sigma-Aldrich), water removed under vacuum, and the residue dissolved in 1 mL of water. Extract was spotted on TLC plate (Sigma-Aldrich) and developed in acetone/diethyl ether/water (7/3/1, v/v/v). The plates were sprayed thoroughly with 0.2% ninhydrin (Sigma-Aldrich) and allowed to dry at 70°C for 20 min. To confirm the nature of the hexose, the sugar spot was taken from the TLC plate and therefore analyzed by high performance liquid chromatography (HPLC) as described by Sikander et al. (2016). The fatty acids profile of the glycolipid was examined as described previously (Kitamoto et al., 1998). The methyl ester derivatives of the fatty acids were prepared by mixing the above purified glycolipid (10 mg) with 5% HCl-methanol reagent (1 mL). After the reaction was quenched with water (1 mL), the methyl ester derivatives were extracted with hexane and then analyzed by gas chromatography-mass spectrometry (GC-MS) as described before (Hanano et al., 2013; Hanano et al., 2015).

### Determination of the Critical Micelle Concentration (CMC)

The CMC is an important characteristic of a biosurfactant as it is defined the concentration of biosurfactant requisite to form micelle. In order to establish the CMC of the purified biosurfactant isolated from *B. safensis* PHA3, the relationship between the biosurfactant concentration and the surface tension was determined. The freeze-dried purified biosurfactant was dissolved in the distilled water at various concentrations ranged from 0.01 to 8 mg mL^-1^. The CMC was determined by plotting the surface tension as a function of the biosurfactant concentration and was found at the point of intersection between the two lines that best fit through the pre- and post-CMC data. The surface tension of each sample was determined by the Ring method (Gudina et al., 2010) at room temperature (about 23°C). All measurements were done in triplicate.

### Determination of Emulsifying Activity of the Biosurfactant and the Effects of Environmental Conditions

Emulsifying activity of the purified biosurfactant was determined according to Thavasi et al. (2011b). As control each substrate was maintained with above buffer solution without biosurfactant added and the values obtained for each control was subtracted from emulsification values obtained from its respective experiment. The recorded emulsifying activities of the biosurfactant toward the indicated substrates (**Table 4**) were compared with the standard chemical surfactant Triton X-100 by following the same protocol. Measurements for each substrates were performed in triplicate. The effectiveness of the purified biosurfactant to emulsify the PCO at different temperatures, pH and salinities was determined as described before (Chandankere et al., 2013). All experiments were performed in triplicate.

### Antimicrobial Assays

The antimicrobial activity of the purified biosurfactant against several microbial strains was firstly defined by the inhibition zone essay. Bacterial strains were cultured into LB broth while yeast strain was cultured into PD broth for overnight at the appropriate temperature for each strain. One hundred microliters of each overnight culture (DO_600_ ≈ 1) were spread on the surface of solid LB or PD Petri dishes. The pure biosurfactant dissolved in a sterile water (at 1, 5, and 10 mg mL^-1^) was loaded onto a sterile disk filter paper and replaced in the center of the plates. Disks loaded only with a sterile water were used as control for each strain tested. Plates were incubated for 48 h at 37°C for bacteria or at 28°C for yeast. In parallel, the antimicrobial activity was also tested by the microdilution method according to Gudina et al. (2010) with few modification. Briefly, an initial cells count equal to 10^4^ CFU mL^-1^ was used for each strain. The biosurfactant, dissolved in water, was added directly to the culture medium at 1, 5, and 10 mg mL^-1^ as final concentrations. Cultures with their respective control were incubated for 48 h at 37°C for bacteria or at 28°C for yeast. Forty-eight hours later, the CFU mL^-1^ was determined for each culture and its respective control. Triplicate assays were performed at all the biosurfactant concentrations for each strain.

### Molecular and Transcriptional Analysis of Biosurfactant Biosynthesis Gene

The genome sequencing of few strains of *B. safensis* revealed that this species harbors a gene that encodes for a key enzyme catalyzing the biosynthesis of glycolipids, known as a diacylglycerol glucosyltransferase or as a β-DGS (Satomi et al., 2006). Multi-alignment of the amino acid sequences of β-DGS showed that these proteins are highly conserved in various bacterial species (Supplementary Figure S1). We therefore designed β-DGSF and β-DGSR, gene-specific primers to amplify the full length ORF of β*-DGS* gene using http://www.bioinformatics.nl/cgi-bin/primer3plus/primer3plus.cgi (Supplementary Table S1). The bacterial genomic DNA was extracted and used as a template for PCR amplification as described before (Hanano et al., 2014). The PCR amplicon was analyzed on a 1.5% agarose gel then cut off, extracted, and purified using a QIAquick PCR purification kit (Qiagen, Germany) as described in the product handbook. PCR products were sequenced on an ABI 310 Genetic Analyzer (Applied Biosystems). The obtained nucleotide sequence was compared with database sequences using Blastn provided by NCBI^1^. The sequence generated in this study was subsequently deposited in the GenBank database under the accession number KU507541. The predicted amino acid sequence was multi-aligned with other bacterial proteins and clustered using http://www.phylogeny.fr/. Transcriptional analysis of β*-DGS* gene was evaluated as a function of the nature of carbon sources as described before (Hanano et al., 2013).

### Analysis of PAHs and *n*-Alkanes Degradation

For the degradation study, the bacterial strain was inoculated in MSM containing a PAH (1 mM). The different compositions used in the degradation of PAH were (i) medium+PAH+bacterial strain; (ii) medium+PAH; and (iii) medium+bacterial strain, where (ii and iii) served as controls. Approximately 1 × 10^4^ CFU mL^-1^ of *B. safensis* PHA3 was added to the medium as an initiating bacterial cell count. The cultures, prepared in triplicate, were incubated at 28°C on a rotary shaker at 200 rpm and the degradation of PAHs was followed every day for 7 days by extracting and analyzing of the residual PHAs as described previously (Arulazhagan and Vasudevan, 2011). In brief, PAHs were extracted twice with an equal volume of ethyl acetate after acidification to pH 2.5 with 1 N HCl. The extracts were filtered through anhydrous sodium sulfate and evaporated until dryness under a flow of nitrogen. The extract was resuspended with 100 μL acetonitrile and stored in an amber-colored vial under refrigeration. Extracts were analyzed using a Jasco LC-2000 plus series HPLC system (Jasco, USA) using a UV-detector at 245 nm and a Vydac 201TP54 column (250 mm × 4.6 mm, 5 μm particles, Agilent, USA). The analysis was performed using a mobile phase of acetonitrile/water (80/20, v/v) at a flow rate of 1 mL min^-1^ and a run time of 25 min. Standard solutions of different PAHs were used as a reference. The samples were injected individually and the residual amount of PAHs was calculated based on the peak area. Oxidation of *n*-alkane by *B. safensis* PHA3 was determined according to Hanano et al. (2015). The bacterial strain was cultured as described before in the presence of a sole and specified amount (1 mM) of *n*-octadecane (*n*-C18) and *n*-tetracosane (*n*-C24) separately. The cultures were shaken in an orbital shaker at 200 rpm and 28°C for 1 h. After the extraction of the residual substrate and products by vigorous mixing with 1 mL of ethyl acetate, the samples were centrifuged at 5.000 × *g* for 5 min. The ethylacetate layer was harvested, dried with Na_2_SO_4_, stored in a capped GC vial. Analyses by GC-MS with H_2_ as carrier gas at a flow rate of 2 mL min^-1^ were performed on an Agilent 6850 gas chromatograph as previously described (He et al., 2005).

### Effect of Biosurfactant on the PCO-Biodegradation

Shake flask biodegradation experiments were carried out in 500 mL Erlenmeyer flasks with 250 mL of MSM according to Thavasi et al. (2011b). Prior to inoculation, various concentrations of purified biosurfactant were pre-added to the medium. Then the culture medium was inoculated with 1% (v/v) inoculum containing 10^4^ bacterial cells mL^-1^ and the culture flasks were maintained in a shaker for 6 days. The effect of isolated biosurfactant on the biodegradation of PCO were evaluated in three different experimental sets; Set I: bacterial cells in MSM+crude oil (normal), Set II: bacterial cells in MSM+biosurfactant+crude oil, and Set III: free MSM+crude oil (No bacterial cells). The concentrations of biosurfactant used in the Set II were 0.05, 0.1, 0.15, 0.2, 0.25, 0.3, 0.35, 0.4, 0.45, and 0.5% (w/v). Crude oil degradation was estimated fluorometrically as described in the IOC protocols. (Intergovernmental Oceanographic Commission Manuals and Guide No. 13, 1982). Five milliliters of culture medium was taken from each experimental sets and centrifuged at 6000 rpm to remove the bacterial cells. Crude oil residues from the supernatant were extracted with an equal volume of hexane. Crude oil content in the hexane extract was measured in a fluorescence spectrophotometer at 310 nm excitation and at 374 nm emission wavelengths. Values were compared with a standard curve established with different concentrations of crude oil and the degradation levels were expressed as percentage. Degradation values from the non-inoculated control flask (Set III) was treated as natural weathering of crude oil and it was estimated as 5% and the value was subtracted from the results obtained in other experimental sets (Set I and II). All the experiments were done in triplicate and the mean values were used as results.

### Statistical Analysis

All the data are expressed as mean ± standard error mean (SEM). Experimental data were analyzed by two-way analysis of variance (ANOVA) followed by Tukey–Kramer multiple comparisons test. Statistical difference yielding *p* < 0.05 were considered significant.

## Results

### Isolation and Molecular Identification of the PCO-Dwelling Bacterial Strains

A PCO-inhabiting bacterium was isolated by applying enrichment culture technique using a MSM. Based on this strategy, seven bacterial strains were therefore purely isolated and referred as PHA2, PHA3, PHA5, PHA6, PHA7, PHA8, and PHA9. The bacterial strains were differentiated by their colony color, Gram staining, spore-forming and by their cell morphology under light microscopy (Supplementary Table S2). We therefore embarked to molecularly identify these strains by amplifying and sequencing a fragment of their respective *16S rRNA* genes. Blasting of the obtained *16S rRNA* sequences against the public NCBI-databases showed a high homology, with a score more than 99%, with other *16S rRNA* genes identified in *Bacillus* sp., *Planomicrobium* sp., *Psychrobacter* sp., *Streptomyces* sp., and *Kocuria* sp. The isolate names, length of *16S rRNA* gene fragments, identity of bacterial species, their respective GenBank accession numbers and their major characteristics with related references are presented in **Table 1**. All seven bacterial strains were evaluated in the respect of their capabilities to grow in the presence of crude oil as a sole carbon source.

### *Bacillus safensis* PHA3 Differentiates by Its Capability to Use the PCO

**FIGURE 1:**
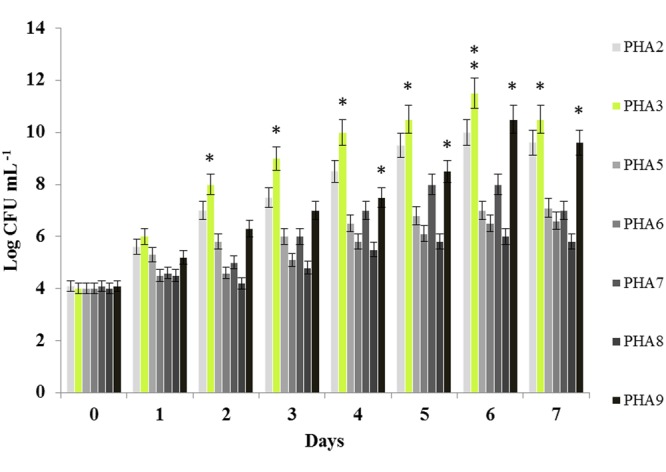
**Bacterial cell count in the presence of PCO as a sole carbon source.** Each bacterial isolate was cultured in MSM medium containing 2% of PCO for 7 days at 28°C. The bacterial growth was expressed as Log CFU mL^-1^. The experiments were performed in triplicate and the presented data are mean values ± SD. Asterisks indicate significant differences: ^∗^*P* < 0.05 (significant); ^∗∗^*P* < 0.01 (very significant).

All seven bacterial isolates were evaluated by their PCO-assimilation activities. For this purpose, the cells count, expressed as Log CFU mL^-1^, of the bacterial strains in the presence of PCO as a sole carbon source were compared. As shown in **Figure 1**, bacterial cell count revealed that all seven bacterial strains differentially utilized the PCO, which was evident from the maximum cell count observed after 7 days of incubation. The three highest values of the bacterial cell growth were remarkably observed for *Bacillus safensis* PHA3, *Kocuria flava* PHA9 and *Bacillus pumilus* PHA2 where their cells account reached 11.5, 10.5, and 10 Log CFU mL^-1^ (**Figure 1**). Overall, the cell count for all seven strains in shake flask experiments was ordered as following: *Bacillus safensis* PHA3 > *Kocuria flava* PHA9 > *Bacillus pumilus* PHA2 > *Bacillus licheniformis* PHA7 > *Planomicrobium* sp. PHA5 > *Psychrobacter* sp. PHA6 > *Streptomyces champavatii* PHA8. Based on this primary evaluation, the strain *B. safensis* PHA3 was differentiated by its capability to use the PCO and it was therefore subjected for further biochemical and molecular characterizations.

### SEM Imaging and Phylogenetic Analysis of *B. safensis* PHA3

**FIGURE 2:**
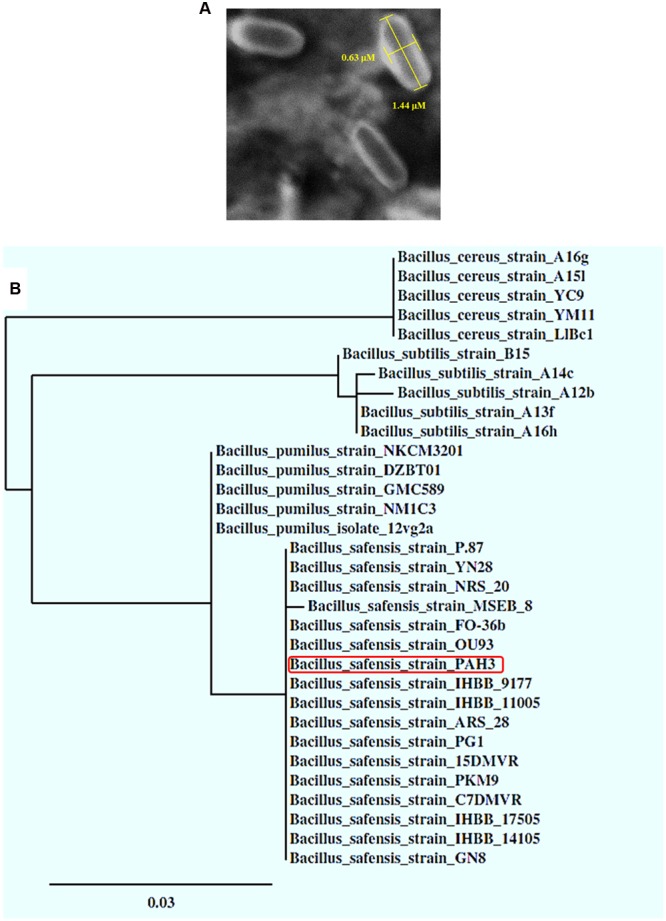
**SEM imaging and phylogenetic analysis of the *B. safensis* PHA3. (A)** Scanning Electron Microscope (SEM) imaging of *B. safensis* PHA3. **(B)** Phylogenetic relationship between the stain *B. safensis* PHA3 and other strains or species based on the homology of *16S rRNA* sequences. The identified strain PHA3 is indicated by a red rectangle.

Molecularly identified as *Bacillus safensis* PHA3, this species is known by its small size cells (Satomi et al., 2006). The observation of bacterial cells under light microscope, at the magnification of 100×, was not informative (data not shown). Thus, the examination of bacterial cells under a SEM showed their rod-shaped forms with cell size ranged from 0.5 to 0.7 μm in diameter and 1.4 to 1.7 μm in length (**Figure 2A**). Moreover, phylogenetic analysis, based on the homology of *16S rRNA* genes, revealed that the strain PHA3 was in all probability clustered in the same branch of *B. safensis* species and even more located at the same distance of many other strains identified under this species, i.e., FO-36b, OU93, IHBB9177, and IHBB11005 (**Figure 2B**). Such morphological and phylogenetic analysis confirm the molecular identity of PHA3 and strongly suggest that this isolate is a new strain of *B. safensis.*

### *Bacillus safensis* PHA3 Produces an Efficient Biosurfactant

**FIGURE 3:**
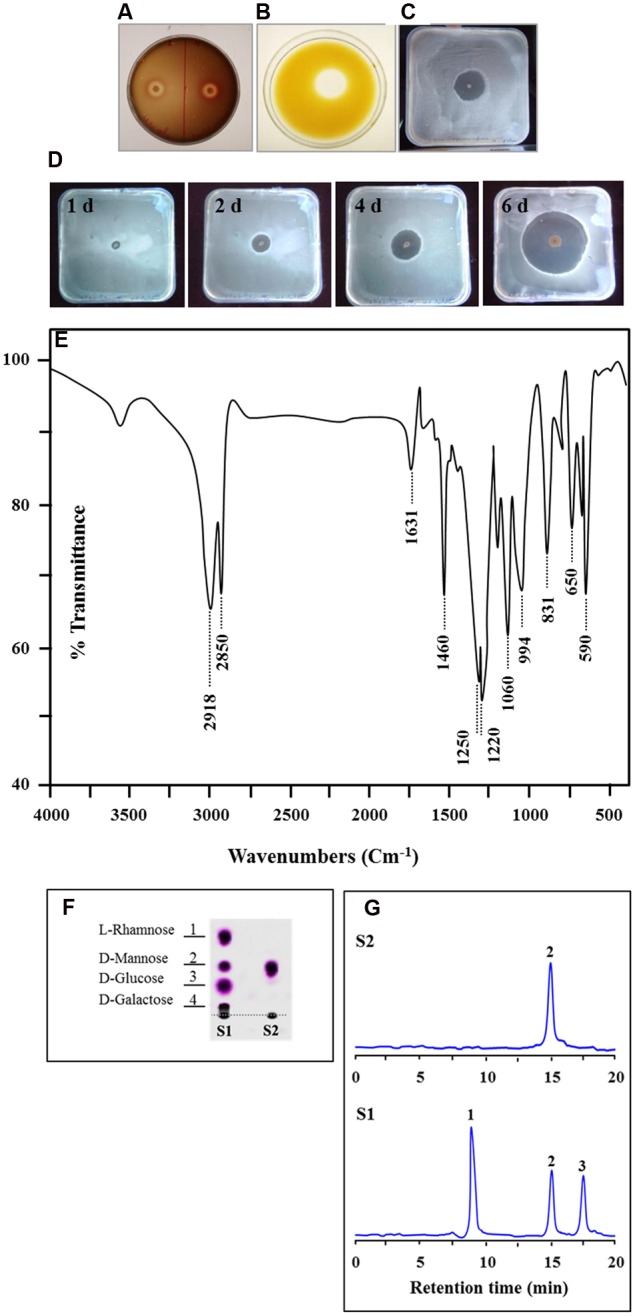
**Screening and characterization of the biosurfactant produced by *B. safensis* PHA3.** The capability of bacteria to produce biosurfactant was primarily shown by the hemolytic activity **(A)**, by the oil displacement method **(B)**, and by the hydrocarbon overlay agar test **(C)**. **(D)** Development of the hydrocarbon escaping zone on agar plates overtime. **(E)** FT-IR spectra of the biosurfactant produced by *B. safensis* PHA3. **(F)** Thin layer chromatography (TLC) for the hydrolysis sugars of the biosurfactant (S2) compared with a mixture of standard sugars (L-Rhamnose, D-Mannose, D-Glucose and D-Galactose) (S1). **(G)** HPLC chromatograph of the hydrolysis hexose of the biosurfactant (peak 2, panel S2) compared with standards (peak 1; L-Rhamnose, peak 2; D-Mannose and peak 3; D-Glucose, panel S1). All experiments were done in triplicate.

The capability of *B. safensis* PHA3 to produce biosurfactant molecules was primarily screened by the hemolytic activity, the oil displacement method, the HOA test and the BATH essay. *B. safensis* PHA3 showed positive results for all of such screening assays. An exceptional hemolytic activity (5 mm), a remarkable oil displacement capability (10 mm) and an interesting hydrocarbon escaping zone (10 mm) were revealed by *B. safensis* PHA3 (**Figures 3A–C**). Moreover, the production of biosurfactant by *B. safensis* PHA3 was progressively increased over time, the maximum hydrocarbon escaping zone (22 mm) was measured on day 6 (**Figure 3D**). Altogether, these results suggest that *B. safensis* PHA3 produces an effective biosurfactant. This biosurfactant was therefore extracted, purified and biochemically characterized.

### The Biosurfactant Produced by *B. safensis* PHA3 Is a Glycolipid

The biosurfactant was successfully extracted by acid precipitation from the supernatant of 7-day-old cultures. Based on the biochemical analysis of carbohydrate, lipid, and proteins, the resulting extract consisted of carbohydrate and lipid with a combination of 32:68 (carbohydrate/lipid; w/w) and was free of proteins. Furthermore, the molecular composition of the extracted biosurfactant was confirmed by Fourier Transform Infrared spectroscopy (FT-IR). The recorded FT-IR-spectrum, which covered region from 500 to 4000 cm^-1^, is depicted in **Figure 3E**. The FT-IR-spectrum revealed that the most important bands were located at 2918 and 2850 cm^-1^ (for the C-H aliphatic stretching), 1631 cm^-1^ (C-O stretching vibrations of carbonyl group), 1061 cm^-1^ (PII band: polysaccharides), and 831, 650 cm^-1^ (for the CH_2_ group). The spatial arrangement of proton and carbon in the biosurfactant molecule, determined by ^1^H NMR and ^13^C NMR spectroscopy, revealed that the chemical shifts were typically similar to the molecular structure of a glycolipid. Assignments of chemical shift presented in **Table 2** indicated to C1, C2, C3, C4, C5, C6, OCH3, and –COOH groups in ^1^H and ^13^C NMR spectra. These NMR peaks seemed to be characteristic to a glycolipid molecule with two distinguished moieties, a hexose and a fatty acid chain.

**Table 2:** Chemical shift assignment (NMR data) of the biosurfactant produced by *B. safensis* PHA3.

Assignment(s)	^1^H NMR (ppm)	^13^C NMR (ppm)
C1	5.1	91.6
C2	3.63	71.4
C3	3.82	72.8
C4	3.91	71.6
C5	3.53	69.4
C6	3.92, 3.81	60.8
OCH_3_	3.52	53.41
COOH	–	170.59

Next, the biosurfactant was subjected to acid hydrolysis to determine the nature of hexose moiety. The qualitative analysis on the TLC revealed that the fraction of acid hydrolyzate from biosurfactant contained a unique spot with a similar *R_f_* to D-Mannose compared with a mixture of standard sugars, l-Rhamnose, D-Mannose, D-Glucose and D-Galactose (**Figure 3F**). The unique spot which had a *R_f_* (0.57) was taken and further analyzed by HPLC. The HPLC chromatograph for the hexose moiety of the biosurfactant depicted the presence of a sole peak (peak 2) at a retention time of 14.9 min (**Figure 3G**, panel S2). The peak 2 is perfectly superposed to the peak corresponding to D-Mannose of standards run (**Figure 3G**, panel S1). These data suggest that the hexose moiety of the extracted biosurfactant is likely being a D-Mannose.

Furthermore, the fatty acid composition of the purified biosurfactant was determined by GC-MS and compared with their respective methyl esters standards. Analysis data presented in **Table 3**, showed that the lipid moiety of the biosurfactant is majorly comprised from Hexadecanoic acid (16:0) with 86.4 ± 4.0% of the total fatty acids. In addition, Octadecanoic acid (18:0) and Tetradecanoic acid (14:0) were found at low percentages, 10.3 ± 0.8 and 4.1 ± 0.2, respectively. Altogether, these data are quite informative to give strong indication that the extracted biosurfactant under investigation is likely a mannolipid molecule with a hydrophilic part, the D-Mannose, and a hydrophobic part, the Hexadecanoic acid.

**Table 3:** Fatty acids composition of the biosurfactant produced by *B. safensis* PHA3.

Fatty acids composition (% ± SD)	
12:0	UD
14:0	4.1 ± 0.2
16:0	86.4 ± 4.0
18:0	10.3 ± 0.8
18:1	UD
18:2	UD
20:0	UD
22:1	UD

*UD, Undetectable.*

### *Bacillus safensis* PHA3 Is a Potential Producer of Biosurfactant

**FIGURE 4:**
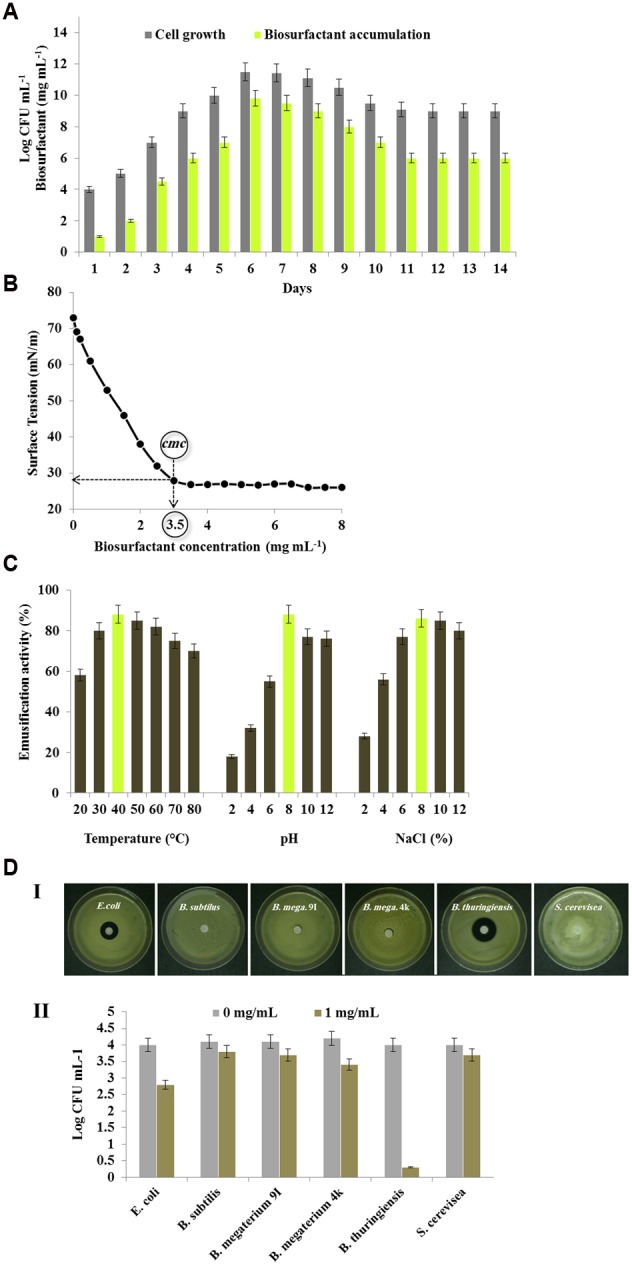
**Production and potential of the biosurfactant produced by *B. safensis* PHA3. (A)** Time course of the biosurfactant production by the strain PHA3 grown into MSM medium supplemented with 2% PCO (v/v). **(B)** Shake flask-biodegradation experiments of PCO in the presence of various concentration of the purified biosurfactant. **(C)** Effects of temperature, pH and salinity on the emulsification activity. **(D)** The antimicrobial activity of the purified biosurfactant against several microbial strains was firstly defined by the inhibition zone essay (I) then tested by the microdilution method (II). All experiments were done in triplicate and the presented data are mean values ± SD.

After having determined the biochemical nature of the biosurfactant produced by *B. safensis* PHA3, its production was evaluated over time and as a function of the bacterial cell growth. **Figure 4A** shows the time course of the biosurfactant production by *B. safensis* PHA3 grown in the presence of PCO as a sole carbon source. The maximum concentration of the biosurfactant, about 9.8 ± 0.5 mg mL^-1^, was measured on day 6, when the cell growth reached its early stationary phase. In parallel, the maximum cell count was observed on the same day (11.6 Log CFU mL^-1^). Then, the production of biosurfactant briefly declined on day 7 to day 10 and stabilized between 11 and 14 days.

The CMC is an important characteristic of a biosurfactant as it is defined the biosurfactant concentration requisite to form micelle. In order to establish the CMC of the purified biosurfactant, the relationship between the biosurfactant concentration and the surface tension was determined. The freeze-dried purified biosurfactant was dissolved in a distilled water at various concentrations from 0.01 to 10 mg mL^-1^. As shown in **Figure 4B**, the distilled water was found to have a surface tension of 73 mN/m and the addition of biosurfactant reduced its surface tension to 26 mN/m. Upon reaching the CMC (3.5 mg mL^-1^) the surface tension became stable, and no significant reductions were detected even at the highest concentrations tested.

Moreover, emulsification of various hydrophobic substrates by the *B. safensis* PHA3-biosurfactant was compared with Triton X-100, an excellent dispersant and chemical emulsifier for oil-in-water systems. The data of emulsifying activities, expressed as a OD_610_, showed that the purified biosurfactant emulsified all used substrates with activity values equivalent to those obtained by Triton X-100 (**Table 4**). The highest emulsifying activity of the biosurfactant was measured against crude oil followed by cotton oil, olive oil, and peanut oil. The biosurfactant moderately emulsified diesel and kerosene and less the naphthalene. The results clearly indicate that the purified biosurfactant produced by *B. safensis* PHA3 exhibits a high emulsifying activity.

**Table 4:** Emulsifying activity of the purified biosurfactant from *B. safensis* PHA3 compared with Triton X-100.

Substrate	Emulsifying activity (OD_610_)	
	*B. safensis* -biosurfactant	Triton X-100
Crude oil	1.95 ± 0.1	1.94 ± 0.1
Diesel	1.20 ± 0.05	0.95 ± 0.1
Kerosene	1.15 ± 0.1	1.25 ± 0.1
Naphthalene	0.46 ± 0.05	0.64 ± 0.1
Peanut oil	1.66 ± 0.1	1.55 ± 0.1
Olive oil	1.74 ± 0.1	1.68 ± 0.1
Cotton oil	1.80 ± 0.1	1.84 ± 0.1

Further, an effective and promising emulsifier should show a high activity over different physico-chemical conditions. In this purpose, the emulsification activity (EA) of the *B. safensis* PHA3-biosurfactant toward the PCO, expressed as OD_610_, was tested over a wide range of variation in temperature, pH, and salinity. Firstly, the PCO-emulsifying activity manifested highest values when the biosurfactant was pre-incubated at temperature between 30 and 60°C (EA < 85%). Whilst these values declined when the biosurfactant was heated at more than 70°C or cooled at less than 20°C (**Figure 4C**). Regarding the pH effect, the PCO-emulsifying activity was optimal (EA = 88%) at pH 8 then briefly decreased in alkaline conditions. However, in acidic conditions (pH 2, 4, and 6) the PCO-emulsifying activity was seriously affected and did not exceed 18, 32, and 55%, respectively (**Figure 4C**). Finally, the PCO-emulsifying activity of biosurfactant (EA = 86%) was optimal at a briefly high salinity 8% NaCl. Whilst a low salinity conducted a low PCO-emulsifying activity (**Figure 4C**). It is still to note that the *B. safensis* PHA3-biosurfactant did not precipitate at high concentration of NaCl.

Finally, one of the most critical properties of a biosurfactant is its antimicrobial activity. To evaluate that, the antimicrobial activity of the purified biosurfactant was tested against a groups of non-pathogenic microorganisms including few bacterial strains, i.e. *Escherichia coli, Bacillus subtilis, Bacillus megaterium* 9I, *Bacillus megaterium* 4k, *Bacillus thuringiensis*, and a yeast strain of *Saccharomyces cerevisiae*. The panel I of **Figure 4D** shows a visible inhibitory effect of the biosurfactant on the growth of some bacterial strains notably, *E. coli* and *B. thuringiensis*. While no remarkable effect was observed for the biosurfactant against other bacterial strains and the yeast *S. cerevisiae* SHSY. Such inhibitory effect was confirmed in liquid culture in the presence of various concentration of the biosurfactant. The cells count, expressed as Log CFU ml^-1^, for the bacterial and yeast strains grown in the presence of the biosurfactant revealed that such glycolipid decreased dramatically the cells count of *B. thuringiensis* from 4 to 0.3 Log CFU mL^-1^ after 48 h of incubation (**Figure 4D**, panel II). Less decrease was detected in cells count of *E. coli* and *B. megaterium* 4k (2.8 and 3.4 Log CFU mL^-1^, respectively). Whereas, the growth of other microorganisms was not affected by the addition of biosurfactant. The antimicrobial activities against the microorganisms did not change when the concentration of biosurfactant was augmented to 5 or 10 mg mL^-1^ (data not shown). Altogether, these results show a selective antimicrobial activity for the isolated biosurfactant against few bacterial isolates.

### Molecular and Transcriptional Analysis of β*-DGS-*Encoding Gene in PHA3

**FIGURE 5:**
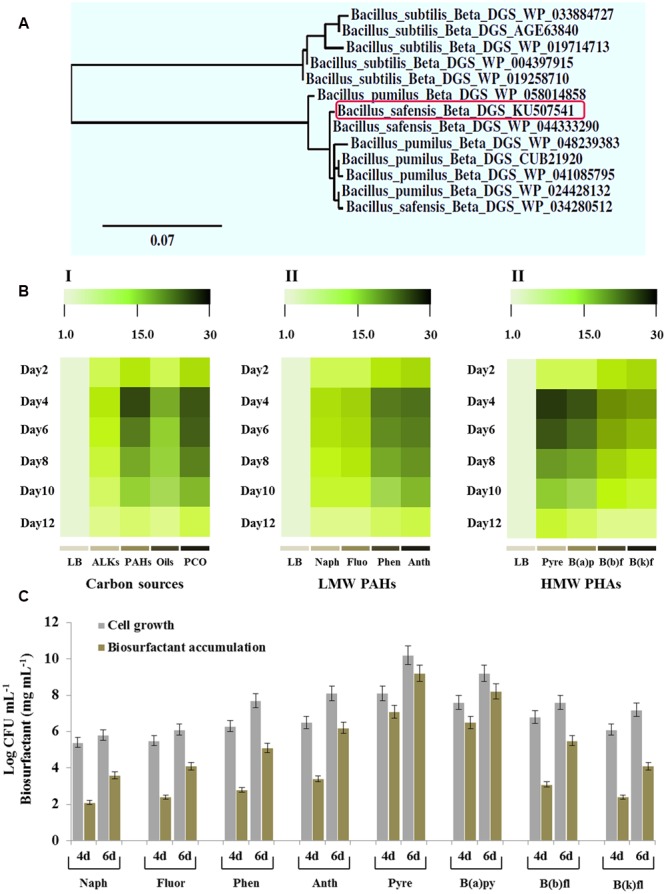
**Phylogenetic, transcriptional analysis of the β*-DGS*-encoding gene and accumulation of the biosurfactant and bacterial cell count as a function of various kinds of PAHs. (A)** Phylogenetic relationship between the protein β-DGS of *B. safensis* PHA3 and other orthologs. The newly identified β-DGS (KU507541) is indicated by a rectangle. **(B)** Transcripts levels of the β*-DGS*-encoding gene in *B. safensis* PHA3 as a function of carbon sources. The color scale (white–green–black) indicates relative changes of transcript abundance of 1-, 10-, and 20-fold, respectively. **(C)** Accumulation of the biosurfactant and the bacterial cell count as a function of various kinds of LMW-PAHs and HMW-PAHs on days 4 and 6 after inoculation. All experiments were done in triplicate and the presented data are mean values ± SD.

The genome sequencing of few strains of *B. safensis* revealed that this species harbors a gene encoding a key enzyme catalyzing the biosynthesis of glycolipids. This enzyme has been previously identified as a diacylglycerol glucosyltransferase also known as a β-DGS (Satomi et al., 2006). Using the gene homology approach, we identified an orthologous β*-GDS* gene in the genome of *B. safensis* PHA3. The sequencing of the full-length gene revealed that its Open Reading Frame (ORF) is composed of 1149 bp and encodes a protein of 383 amino acids. The deduced amino acid sequence of this ortholog, submitted to NCBI under the accession number KU507541, shared a high identity (more than 97%) with other β*-DGS* proteins identified in various strains of *B. safensis, B. pumilus*, and *B. subtilis* (Supplementary Figure S1). Phytogenic analysis, based on the protein sequences homology indicates that the β-DGS of *B. safensis* PHA3 is clustered at the same distance from a β-DGS (WP_044333290) isolated from *B. safensis* (**Figure 5A**).

As pointed out before, the bacterial glycolipid was only produced when bacteria was grown in the presence of PCO as a sole carbon source, we evaluated therefore the expression of β*-DGS* gene as a function of the hydrocarbon sources. Thus, the panel I in **Figure 5B** shows that β*-DGS* gene was more highly expressed in the presence of hydrocarbons compared with LB culture. As a function of the chemical nature of carbon source, the expression levels of the target gene were effectively regulated. On day 4, the expression of β*-DGS* gene was significantly and remarkably induced by a mixture of PAHs and PCO (about 28- and 20-fold, respectively) but less by ALKs and vegetable oils (**Figure 5B**, panel I). Subsequently, various kinds of PAHs were tested in the respect of their activity in inducing of the β*-DGS* expression. A representative group of low molecular weight (LMW) PAHs, such as naphthalene, fluorene, phenanthrene, and anthracene, differentially induced the expression of β*-DGS*. The transcript level of β*-DGS* was briefly induced by naphthalene and fluorene (8- to 15-fold, respectively) and more by phenanthrene and anthracene (20- to 22-fold, respectively) (**Figure 5B**, panel II). However, pyrene, benzo(e)pyrene, benzo(b)fluoranthene and benzo(k)fluoranthene, classified as high molecular weight (HMW) PAHs, caused the highest inductions where the gene transcripts were increased about 28- to 26-fold in the presence of pyrene and benzo(e)pyrene, respectively (**Figure 5B**, panel III). These data suggest that PAHs, in particularly pyrene and benzo(e)pyrene, induced the expression of β*-DGS* in *B. safensis* and this induction was peaked on days 4 to 6.

Moreover, the differential peak for the accumulation of β*-DGS* transcripts in the presence of certain PAHs coincided with an optimal growth of the bacteria and a maximal accumulation of the biosurfactant. **Figure 5C** shows that the bacterial growth and the biosurfactant produced increased progressively when bacteria was grown on naphthalene, fluorene, phenanthrene and anthracene and peaked on day 6 using pyrene as substrate (Log CFU ml^-1^ = 10.1 and 9.2 mg mL^-1^ of biosurfactant produced). Taken together, these results indicate that the expression of β*-DGS* gene and the subsequent accumulation of the biosurfactant in *B. safensis* PHA3 is specifically induced by hydrocarbons and more particularly by PAHs that composed from four to five rings.

### *Bacillus safensis* Preferentially but Differentially Degrades the Polycyclic Aromatic Hydrocarbons

**FIGURE 6:**
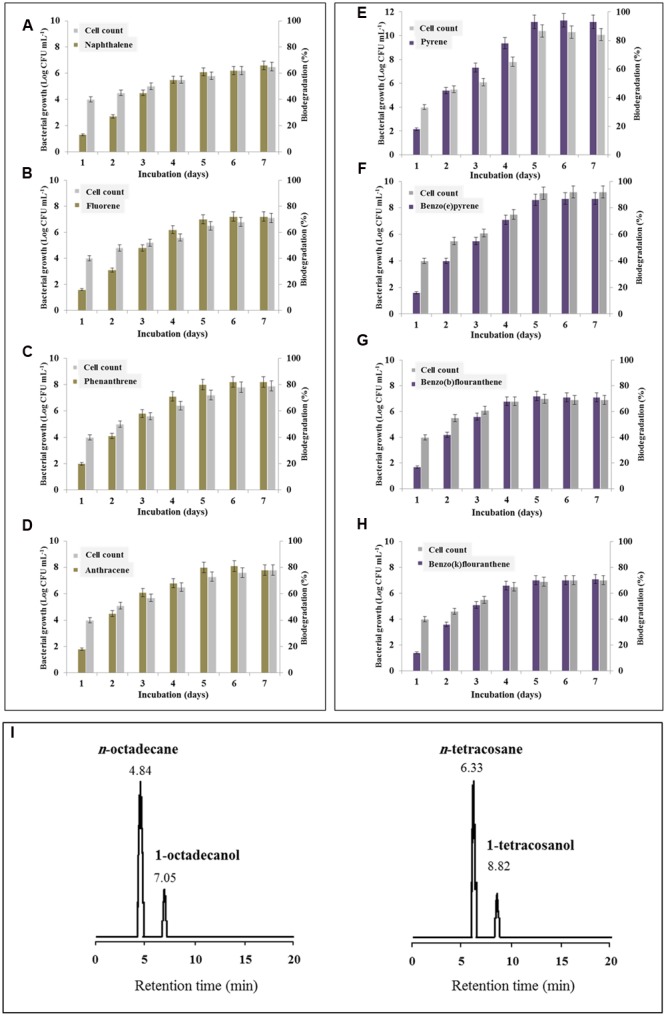
**Degradation of hydrocarbons by *B. safensis* PHA3 in laboratory scale experiments. (A–D)** Degradation of LMW-PAHs, i.e., naphthalene, fluorene, phenanthrene, and anthracene. **(E–H)** Degradation of HMW-PAHs, i.e., pyrene, benzo(e)pyrene, benzo(a)fluoranthene and benzo(k)fluoranthene. The degradation of each PAH was followed individually every day for 7 days by extracting and analyzing of the residual PHA by HPLC. **(I)** GC-MS analysis of products formed by the oxidation of *n*-octadecane and *n*-tetracosane catalyzed by *B. safensis* PHA3 as described in Section “Materials and Methods.” The retention time (min) is indicated for each peak. All experiments were done in triplicate and the presented data are mean values ± SD.

The accumulation of β*-DGS* transcripts and therefore the massive production of the biosurfactant in the presence of certain PAHs raises the question about the biological capability of *B. safensis* in degrading such types of hydrocarbons. The degradation of LMW-PAHs by *B. safensis* PHA3 was therefore demonstrated by the increase of bacterial cell count which paralleled to the percent degradation of the PAHs (**Figures 6A–D**). The residual amount for each type of LMW-PAHs studied was separately analyzed by HPLC and correlated with the bacterial cell count for a period of 7 days. Our results revealed that all LMW-PAHs used as substrates were degraded by *B. safensis* with different efficiency and the optimal degradation value was peaked on day 6. The lowest degradation rate was measured for naphthalene and fluorene (62 and 72%, respectively) while the highest one was estimated for phenanthrene and anthracene (82 and 81%, respectively) (**Figures 6A–D**). From the HMW-PAHs, pyrene and benzo(e)pyrene were the most degraded by *B. safensis* PHA3 (94 and 87%, respectively). Whilst, benzo(a) and benzo(k)fluoranthene were less degraded (about70% for both PAHs) (**Figures 6E–H**).

Otherwise, the brief up-regulation of β*-DGS* by ALKs (**Figure 5B**, panel I) raises the question about the biological ability of PHA3 to consume such hydrocarbons. Thus, the oxidation of *n*-octadecane (*n*-C18) and *n*-tetracosane (*n*-C24) by *B. safensis* PHA3 was followed up by GC-MS. **Figure 6I** shows a brief transformation of *n*-octadecane and *n*-tetracosane (about 12% for both ALKs) into a one major product, the respective *n*-alkyl-alcohol. The corresponding alcohols were identified as 1-octdecanol and 1-tetracosanol with retention times of 7.05 and 8.82 min, respectively (**Figure 6I**). The little transformed amount of both ALKs was correlated to a marginal increase in the bacterial growth (data not shown). Overall, the differential increase in the biodegradation rate for certain PAHs was followed by a remarkable augmentation in the bacterial growth. Altogether these data suggest that *B. safensis* is particularly able to degrade the PAHs and more specifically the pyrene and benzo(e)pyrene. Whilst it shows a brief capability to assimilate the ALKs.

### Finally, the Pre-addition of Glycolipid Enhances the Degradation of PCO by *B. safensis*

**FIGURE 7:**
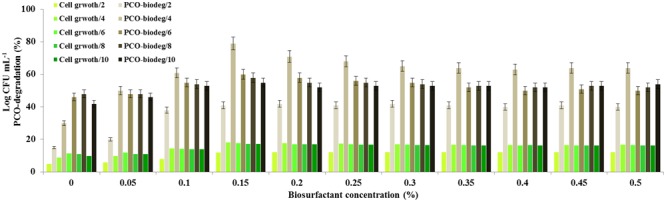
**Enhancement of the degradation of PCO by pre-adding the biosurfactant to the medium.** Shake flask-biodegradation experiments of PCO in the presence of various concentration of the purified biosurfactant. Prior to inoculation, various concentrations of purified biosurfactant were pre-added to the medium. Then the culture medium was inoculated with 1% (v/v) inoculum containing 10^4^ bacterial cells mL^-1^ and the culture flasks were maintained in a shaker for 6 days. Crude oil degradation was estimated fluorometrically as described in the IOC protocols and cell count was determined for each culture. All the experiments were done in triplicate and the mean values were used as results.

In laboratory scale experiments, we investigated whether the pre-addition of the purified biosurfactant to the aqueous medium has any positive effect on the timing and the yield of the PCO-degradation. Among the concentrations used of the biosurfactant, the PCO-degradation was optimal when 0.15% (w/v) was pre-added to the medium (**Figure 7**). At this concentration, the degradation of PCO was enhanced approximately 2.5-fold on day 4 compared with the control (i.e., without biosurfactant). No significant increase in PCO-degradation activity was observed when the biosurfactant was added at concentrations above 0.15%. In parallel, the bacterial cell growth followed this enhancement in PCO-degradation and reached its optimal value (18.2 Log CFU mL^-1^) at 0.15%. Our data indicate that the addition of biosurfactant to the aqueous medium contaminated with PCO enhances the degradation of PCO as well as the bacterial growth on day 4. This means that, at the indicated concentration of biosurfactant, an optimal POC-emulsifying occurs making it more bioavailable to the bacteria and accelerates therefore the biodegradation process.

## Discussion

The contamination of aquatic environments with hydrocarbons is considered one of the most perilous environmental issues. Even though the impressive investigations conducted to validate the use of microorganisms as effective degraders of hydrocarbons (Hamamura et al., 2006; Kanaly and Harayama, 2010; Silva et al., 2014), a particular attention is still paid to identify new microorganisms with new biological horizons in the respect of their degradative activities. Thus, the current study describes the isolation and identification of a set of bacterial strains from the crude oil. Analysis of the *16S rRNA* gene sequences revealed that these strains belonged to different genera, i.e., *Bacillus* sp., *Planomicrobium* sp., *Psychrobacter* sp., *Streptomyces* sp., and *Kocuria* sp. The ability of such bacterial genera to tolerate and therefore to survive in extreme environments has been previously reported. In this context, it was documented that some strains of *B. pumilus* were extremely resistant to environmental stresses (Kempf et al., 2005). Other isolates of *B. safensis* showed a unique tolerance to spacecraft associated environments (Satomi et al., 2006). Some strains of *Planomicrobium* sp. and *Psychrobacter* sp. were known to survive in Antarctic Sea ice and to support an extreme ranges of cold and salinity (Junge et al., 1998; Kim et al., 2012). Furthermore, *Streptomyces* sp. and *Kocuria* sp. were well known as antibiotic producers or aromatic hydrocarbon degraders, respectively (Milind et al., 2001; Zubair et al., 2010).

The evaluation of isolated strains in the respect of their capabilities to grow in the presence of PCO, as a sole carbon source, showed that these isolates differentiated in their activities to use such substrate. This can be supported by earlier reports which indicated that some microorganisms can inhabit and therefore use the crude oil as carbon source (Thavasi et al., 2011b; Chandankere et al., 2013; Hanano et al., 2013; Hanano et al., 2015). More particularly, we found that the strain *B. safensis* PHA3 was the most active consumer of the PCO where it exhibited an optimal value of the bacterial cell growth which is closely similar to the value estimated by Thavasi et al. (2011b), for *B. megaterium*.

Originally, the species *B. safensis* was isolated and identified, for the first time, from a National Aeronautics and Space Administration (NASA) spacecraft assembly facility (Satomi et al., 2006). Thereafter, this species has also been isolated from the saline desert soil (Raja and Omine, 2012; Kothari et al., 2013). More interestingly, it was reported that this species was isolated from biodegraded petroleum samples (Laborda et al., 2014). Therefore, the environmental relevance of this species in biocatalysis and bioremediation makes its genome sequencing an emergent need. Since then, the first *B. safensis* genome was announced in 2013 for a salt-tolerant *B. safensis* strain VK, isolated from saline desert (Kothari et al., 2013). More recently, the genome sequence of a petroleum-degrader strain CFA06 was published (Laborda et al., 2014). The newly gained knowledge on the genetics of *B. safensis* will surely contribute to better understanding its exceptional biological capabilities.

A first and critical challenge to the organisms in the use of hydrophobic organic compounds, such as the PCO, is their potential to insure a competitor level of intracellular hydrophobicity. This can be possible by the production of biosurfactant molecules that tends to reduce the surface tension (Cirigliano and Carman, 1984). The primary screening for the production of such molecules by the strain PHA3, estimated by BATH assay, revealed that the highest cell adherence that found for *B. safensis* cells grown in the presence of PCO was similar to the cell hydrophobicity reported previously by Thavasi et al. (2011b), for *B. megaterium*. In this line, the microorganisms with a high biological capability to modulate their cell surface hydrophobicity are likely more efficient degraders toward hydrocarbons (Hanano et al., 2015).

A detailed and sequential biochemical analysis was performed to determine the composition and the structure of the purified biosurfactant. The recorded FT-IR-spectrum followed by ^1^H NMR and ^13^C NMR spectroscopy permitted to define a primary spatial arrangement of proton and carbon in the biosurfactant molecule. This revealed that the chemical shifts were typically similar to the molecular structure of a glycolipid molecule with two distinguished moieties, a hexose and a fatty acid chain. In line with our analytical data, similar characteristic spectra peaks of NMR were also reported for a Xylolipid from *Enterococcus faecium* and *Lactococcus lactis* (Saravanakumari and Mani, 2010; Sharma et al., 2015) and also for other closely related glycolipids (Morita et al., 2012; Lotfabad et al., 2013). Subsequently, the glycolipid was subjected to acid hydrolysis to determine the nature of hexose moiety as well as the nature of related fatty acids. This conducted to obtain a strong indication that the extracted biosurfactant under investigation is likely a mannolipid molecule with a hydrophilic moiety as mannose and a hydrophobic moiety as hexadecanoic acid. Similar to this, midi-chain saturated fatty acids notably C16:0 and C18:0 were found as main fatty acid chains in various bacterial glycolipids. For example, Sharma et al. (2015), have reported that C16:0 was the main fatty acid which composed the lipid moiety of the glycolipid produced by *Enterococcus faecium*. Moreover, C16:0 and C18:0 were the most abundant fatty acids in the biosurfactant produced by *Lactococcus* sp. (Saravanakumari and Mani, 2010; Vecino et al., 2014). Many earlier reports demonstrated that the biochemical composition of biosurfactant produced by *Bacillus* genus varied according to the species. In line with our results, it was reported that the biosurfactants produced by *B. methylotrophicus* and *B. megaterium* were glycolipids (Thavasi et al., 2008; Chandankere et al., 2013). However, lipopeptides were produced by other species such as *B. subtilis, B. licheniformis*, and *B. mycoides* (Najafi et al., 2010) and even more surfactins were synthesized by *B. substilis* (Nitschke and Pastore, 2006).

Next, after having determined its biochemical nature, we embarked to define the potentiality of the characterized biosurfactant in the respect of the yield, the CMC value, the emulsifying activity, the stability under extreme environmental factors and the antimicrobial activity. In this regard, we found that the highest yield of the biosurfactant peaked on day 6, as a function of the bacterial growth, and this was similar to previous findings reported by Thavasi et al. (2008) on the production of a glycolipid by *B. megaterium*. Beside the yield of the biosurfactant, its CMC value are determinant criteria. So, if we compare these two properties with other bacterial biosurfactants, we found that the yield of biosurfactant produced by *B. safensis* PHA3 was significantly higher compared with earlier reports. It is approximately twofold and sixfold higher than the yield of biosurfactants reported for *B. megaterium* and *Lactobacillus lactis*, respectively, (Rodrigues et al., 2006; Thavasi et al., 2008). Furthermore, the CMC of the isolated biosurfactant is being smaller than earlier values reported in the literature for biosurfactant from other species such as *B. subtilis* and *B. methylotrophicus* (Nitschke and Pastore, 2006; Chandankere et al., 2013, 2014). In addition, a minimum surface tension value of 26.8 mN/m was obtained for the identified biosurfactant which is quite close to the value reported by Chandankere et al. (2014). In general, the efficiency of biosurfactant to reduce the surface tension lower than 30 mN/m is one of the criteria to be considered in selecting an effective biosurfactant. Several lines of evidences confirmed the stability of biosurfactants under extreme thermal, pH and salinity conditions (Batista et al., 2005; Joshi et al., 2008). Comparatively, a similar thermal-pH-salinity profile was reported for a biosurfactant produced by *B. methylotrophicus* USTBa isolated from petroleum reservoir (Chandankere et al., 2013). Regarding the antimicrobial activity, it is well documented that a given biosurfactant may act as an antimicrobial agent against other microbes (Ceresa et al., 2015). Likely, the antimicrobial activity found to be more related to lipopeptides rather than glycolipids (Deepak and Jayapradha, 2015). This last point can be in favor of the biosurfactant isolated in this study. However, a detailed investigation is required to determine the safety of this glycolipid on other bacterial species. Finally, this comparison highlights the potentiality of *B. safensis* PHA3 as an effective producer of biosurfactant and makes it a promising candidate to be used in diverse environmental and industrial applications.

As the production of an effective biosurfactant is a crucial step enabling the microorganisms to initiate the degradation of hydrocarbons, we give a particular attention to study the transcriptional regulation of β*-DGS* gene, a key gene in the biosynthesis of glycolipids. Our findings indicate that the expression of β*-DGS* gene in *B. safensis* PHA3 as well as the accumulation of the biosurfactant are specifically induced by hydrocarbons and more particularly by PAHs suggesting a particular efficient use of this strain as a emulsifier-degrader against such types of hydrocarbons. No comparative data are available regarding the transcriptional analysis of the genes involved in the biosynthesis of bacterial biosurfactants, however, our results can be supported by earlier results showing that the modulation of the biosurfactant biosynthesis in some yeasts is dependent on the growth conditions as well as on many environmental stimuli (Aguilar-Uscanga and Francois, 2003). In this context, it was reported that a considerable increase in the content of the cell wall mannoproteins occurred when the yeast *Saccharomyces cerevisiae* SHSY grew on the PCO and its PCO-emulsification activities were therefore augmented (Hanano et al., 2015).

The accumulation of β*-DGS* transcripts and therefore the massive production of the biosurfactant in the presence of certain PAHs raises the question about the biological capability of *B. safensis* in degrading such types of hydrocarbons. Indeed, our data showed that *B. safensis* PHA3 moderately degraded the LMW-PAHs which composed from two or three aromatic rings. In this line, some reports have demonstrated that more than 70-80% of LMW-PAHs were degraded by halotolerant bacterial strains such as *Ochrobactrum* sp. and *Achromobacter* sp. (Arulazhagan and Vasudevan, 2011; Dave et al., 2014). On the other hand, we found that *B. safensis* PHA3 degraded more actively the pyrene and benzo(e)pyrene, the two first members of HMW-PAHs. However, multiple studies have reported that the HMW-PAHs, with four or more fused aromatic rings are being much more difficult to be biodegraded than the LMW-PAHs (Kanaly and Harayama, 2000). This is due to the high thermodynamic stability and hydrophobicity of HMW-PAHs which make them more adsorbed to solid particles (Talley et al., 2002). In this context, our data suggest that the isolate PHA3, originally isolated from PCO, is more specifically qualified to degrade the pyrene and benzo(e)pyrene. Of particular interest, pyrene has been used as a model compound to study the biodegradation process of HMW-PAHs since it is structurally similar to several carcinogenic compounds (Kanaly and Harayama, 2000). Thus, our findings can be supported by multiple biochemical and molecular evidences that demonstrated the biological capabilities of some bacterial isolates to mineralize pyrene, where the majority of these isolates were identified as species of the genera *Mycobacterium* (Heitkamp et al., 1988; Grosser et al., 1991; Vila et al., 2001; Moody et al., 2004; Zhong et al., 2006; Warshawsky et al., 2007) and *Rhodococcus* (Bouchez et al., 1995). Moreover, our findings revealed a brief assimilation of midi- and long-chain ALKs by the isolate PHA3. This comes in line with earlier results that have shown the ability of *Halomonas eurihalina* strain H-28 to grow and produce biosurfactant on different carbon sources such petrol, *n*-hexadecane, *n*-tetradecane, and crude oil (Martinez-Checa et al., 2002; Pi et al., 2016). Inversely, we previously reported that the microsomes of *Saccharomyces cerevisiae* SHSY as well as the recombinant CYP52A58 preferentially catalyzed the terminal hydroxylation of *n*-hexadecane (C16), followed by *n*-tetracosane (C24), and *n*-hexane (C6) (Hanano et al., 2015). When compared to the above reports, the isolate PHA3 used in the current study degraded LMW-PAHs but more actively HMW-PAHs without any additional substrate. Such characteristics make PHA3 a promising candidate to further molecular characterization of the PAHs-metabolic pathway.

Finally, we demonstrated that the pre-addition of glycolipid to the aqueous medium of PCO effectively accelerated its subsequent degradation by *B. safensis* PHA3. Such enhancement can be explained by the high emulsifying activity of the pre-adding biosurfactant toward the PCO and this makes the hydrocarbons more bioavailable to the bacteria and accelerates therefore their biodegradation process. Comparatively, the values of emulsifying activity, presented in the current study, are higher than the earlier values reported for other PCO-degrading bacteria, *B. megaterium* and *Lactobacillus delbrueckii* (Thavasi et al., 2011a,b). From above, two essential benefits can be taken in consideration. The first is the effectiveness of the isolated biosurfactant compared to the chemically synthesized surfactants, and here, the competitive advantages of the biosurfactants over chemicals should be highlighted, The second is the selectivity of the biosurfactant toward the crude oil, and more particularly its PAHs components. This makes the present biosurfactant a promising agent for multiple environmental and industrial applications.

## Conclusion

An efficient biosurfactant was isolated, purified and characterized from the petroleum-dwelling bacteria, the *B. safensis* PHA3. Biochemically identified as a glycolipid, this biosurfactant (i) is massively produced in the presence of PCO. (ii) It enhanced PCO-degradation when pre-added to the medium at a low concentration. (iii) It is an effective emulsifier against the PCO and vegetable oils. (iv) It maintained an optimal PCO-emulsifying activity in wide-range conditions of temperature, pH and salinity. Moreover, transcriptional and biochemical evidences suggest that PHA3 produces more biosurfactant in the presence of certain PAHs. Subsequently, PHA3 degrades more actively the PAHs but less the midi-chain ALKs. Thus, such remarkable characteristics make the strain *B. safensis* PHA3 a potential model for further studies of the enhanced microbial oil recovery in the aquatic environments.

## Author Contributions

AH led the work, designed all experiments, and wrote the manuscript. IA and MS carried out all experimental work. All authors read and approved the final manuscript.

## Conflict of Interest Statement

The authors declare that the research was conducted in the absence of any commercial or financial relationships that could be construed as a potential conflict of interest.

## Footnotes

1 http://www.ncbi.nlm.nih.gov

## Abbreviations

ALKs: n-alkanes
BATH: bacterial adherence to hydrocarbons
β-DGS: β-diglucosyldiacylglycerol synthase
CMC: critical micelle concentration
NMR: nuclear magnetic resonance
PAHs: polycyclic aromatic hydrocarbons
PCO: petroleum crude oil
