# Supplementary material for: Biochemical, Molecular, and Transcriptional Highlights of the Biosynthesis of an Effective Biosurfactant Produced by Bacillus safensis PHA3, a Petroleum-Dwelling Bacteria
Source: Front Microbiol. 2017 Jan 25;8:77. doi: 10.3389/fmicb.2017.00077 (PMC5263155; doi:10.3389/fmicb.2017.00077)
Supplement: Supplementary file 1 [file Data_Sheet_1.DOC]

**Supplementary Tables**

**Table S1** Primers used in this study.

| **Target gene** | | **Primers** | | **Nucleotide sequence (5’-3’)** | **Amplicon**  **(bp)** | **Reference** |
| --- | --- | --- | --- | --- | --- | --- |
| *16 rRNA* | 27F  1492R | | AGAGTTTGATCMTGGCTCAG  TACGGYTACCTTGTTACGACTT | | 1450 |  |
| *16 rRNA* | Bs16SF  Bs16SR | | GGGACAGAGTGACAGGTGGT  AAGGGGCATGATGATTTGAC | | 179 | This study |
| *β-DGS* | β-DGSF  β-DGSR | | ATGAATACAAATAAAAAAATTCTG  TGACAGTGCTCTTGCATATG | | 1149 | This study |
| *β-DGSq* | β-DGSqF  β-DGSqR | | CCGGATCAACTGAAAGCACT  TAACAATTGCTGCGCCATAA | | 202 | This study |

| **Bacterial strain** | **Gram +/-** | **Spore-forming** | **Cell Morphology** | **Air-dependence** |
| --- | --- | --- | --- | --- |
| PHA2 | + | + | Rod | Aerobic |
| PHA3 | + | + | Rod | Aerobic |
| PHA5 | + | - | Cocci/Rod | Aerobic |
| PHA6 | - | - | Cocci/Diploforms | Aerobic |
| PHA7 | + | + | Rod | Aerobic |
| PHA8 | + | + | Filamentous | Aerobic |
| PHA9 | + | - | Cocci | Aerobic |

**Table S2** Some of general characteristics for the PCO-isolated bacterial strains.

**Supplementary Figures**

**Fig. S1**

**
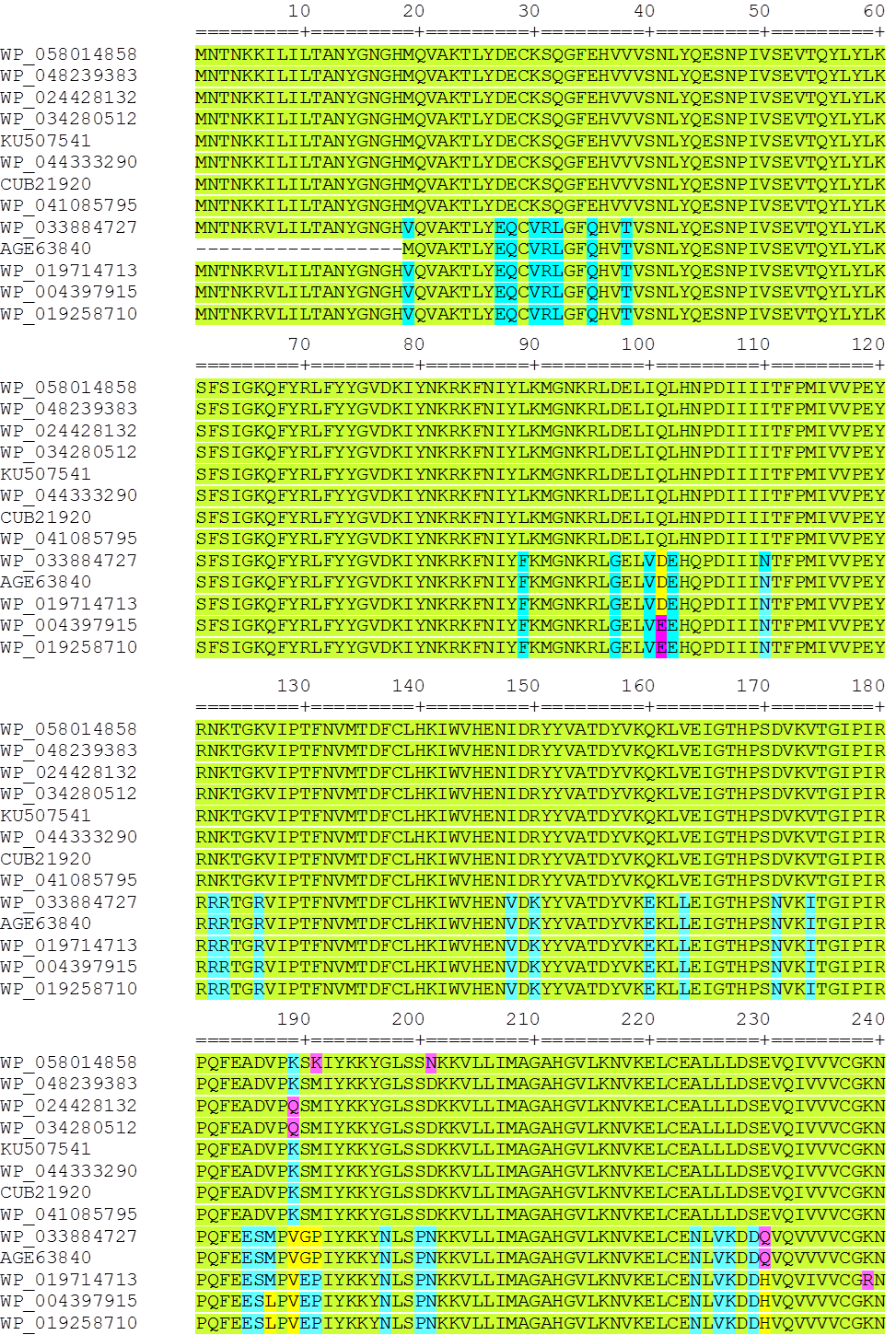
**

**
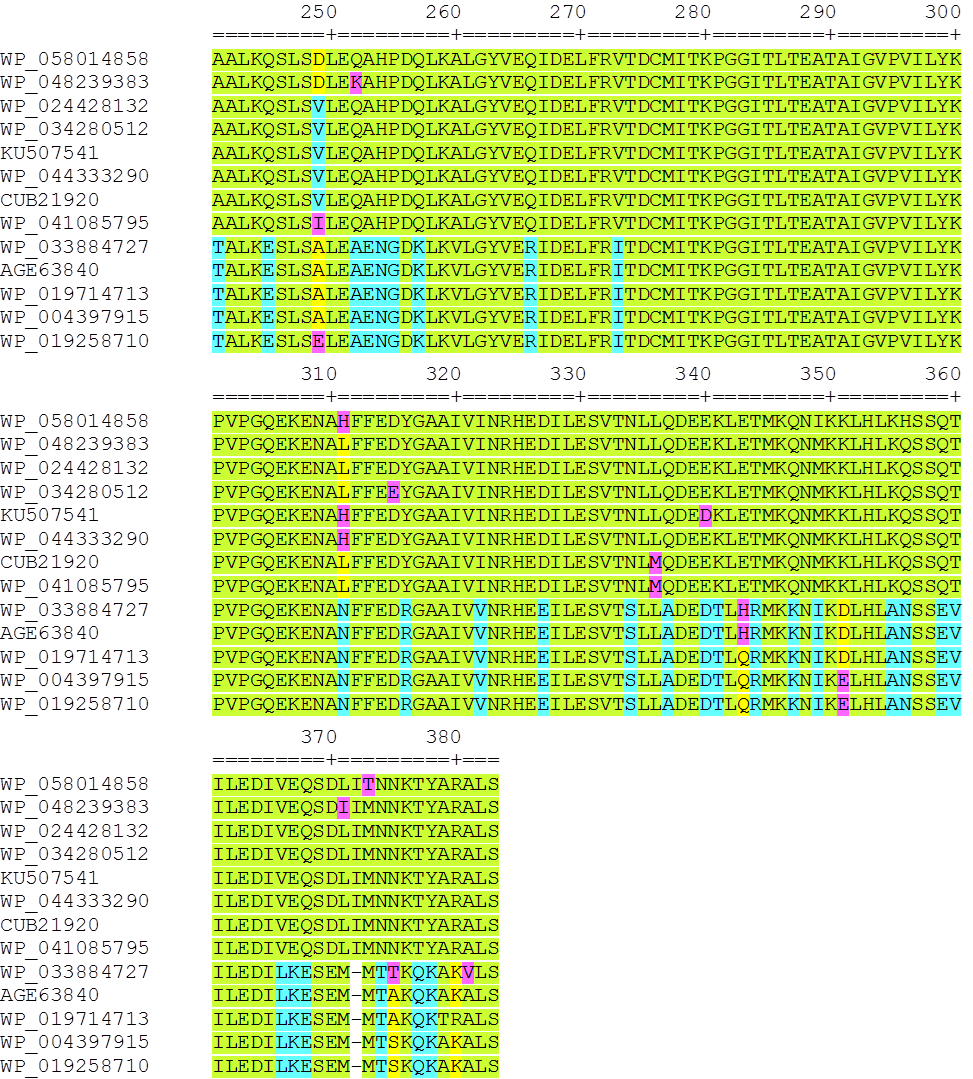
**

**Fig. S1.** Multiple alignment of β-DGS proteins sequences from various bacterial species. Alignment of the β-DGS protein from *B. safensis* PHA3 (KU507541) with others β-DGS identified from *B. safensis* (WP_044333290), *B. safensis* (034280512), *B. pumilus* (WP_058014858), *B. pumilus* (WP_048239383), *B. pumilus* (WP_024428132), *B. pumilus* (CUB21920), *B. pumilus* (WP_041085795), *B. subtilis* (WP_004397915), *B. subtilis* (WP_019258710), *B. subtilis* (WP_019714713), *B. subtilis* (WP_033884727) and *B. subtilis* (AGE63840) was performed with Vector NTI advance version 11.5 (Invitrogen). The Sequence Alignment Pane shows the alignment of the sequences, residues color coded according to identity. The default settings are: Highly identical: Black text on a green background; Moderately identical: Black text on a blue background; Weakly identical: Black on a yellow background; Non-similar: Black text on a pink background.

**Supplementary references:**

Junge, K., Gosink, J.J., Hoppe, H.G., Staley, J.T. 1998. Arthrobacter, Brachybacterium and Planococcus isolates identied from antarctic sea ice brine. Description of Planococcus mcmeekinii, sp. Syst Appl Microbiol, 21, 306-314.

Kempf, M.J., Chen, F., Kern, R., Venkateswaran, K. 2005. Recurrent isolation of hydrogen peroxide-resistant spores of Bacillus pumilus from a spacecraft assembly facility. Astrobiology, 5, 391–405.

Kim, S.J., Shin, S.C., Hong, S.G., Lee, Y.M., Choi, I.-G., Park, H. 2012. Genome Sequence of a Novel Member of the Genus Psychrobacter Isolated from Antarctic Soil. Journal of Bacteriology, 194, 2403.

Lin, X., Lee, C.G., Casale, E.S., Shih, J.C.H. 1992. Purification and Characterization of a Keratinase from a Feather-Degrading Bacillus licheniformis Strain. Applied and Environmental Microbiology, 58, 3271–3275.

Marcial Gomes, N.C., Borges, L.R., Paranhos, R., Pinto, F.N., Mendonca-Hagler, L.C., Smalla, K. 2008. Exploring the diversity of bacterial communities in sediments of urban mangrove forests. FEMS Microbiology Ecology, 66, 96-109.

Milind, W., Rashmi, T., Maithili, J., Bhalachandra, B. 2001. How many antibiotics are produced by the genus Streptomyces ? Archives of Microbiology, 176, 386–390.

Satomi, M., La Duc, M.T., Venkateswaran, K. 2006. Bacillus safensis sp. nov., isolated from spacecraft and assembly-facility surfaces. International Journal of Systematic and Evolutionary Microbiology, 56, 1735–1740.

Zubair, A.R., Nuzhat, A., Geoffrey, M.G. 2010. Isolation of two Kocuria species capable of growing on various polycyclic aromatic hydrocarbons. African Journal of Biotechnology, 9, 3611-3617.
